# Confirmatory non-invasive and non-destructive identification of poison ivy using a hand-held Raman spectrometer

**DOI:** 10.1039/d0ra03697h

**Published:** 2020-06-05

**Authors:** Charles Farber, Lee Sanchez, Dmitry Kurouski

**Affiliations:** Department of Biochemistry and Biophysics, Texas A&M University College Station Texas 77843 USA dkurouski@tamu.edu; The Institute for Quantum Science and Engineering, Texas A&M University College Station Texas 77843 USA

## Abstract

Poison ivy (*Toxicodendron radicans*) is a forest understory plant that grows throughout the United States, Canada and Mexico. The plant contains urushiol oils, a mixture of pentadecylcatechols, that cause severe allergic reactions on skin including reddish inflammation, uncoloured bumps and blistering. Such allergic reactions develop within hours or days, which facilitates unknowing spread of the urushiol inside the house. This enables continuous contact with urushiol extending the length of time of the rash. It should be noted that apart from extensive washing with soap and cold water, there is no direct way to treat urushiol-induced allergic reactions. In these circumstances, the best practice is to avoid contact with the plant. However, differentiating poison ivy from other plants requires sophisticated botanical experience that is not possessed by a vast majority of people. To overcome this limitation, we developed a confirmatory, label-free, non-invasive and non-destructive approach for detection and identification of poison ivy. We show that using a hand-held Raman spectrometer, 100% accurate identification of this species can be performed in only one second. We also demonstrate that in combination with partial least square discriminant analysis (PLS-DA), Raman spectroscopy is capable of distinguishing poison ivy from more than fifteen different plant species, including weeds, grasses and trees. The use of a hand-held spectrometer on a motorized robotic platform or an unmanned aerial vehicle (UAV) can be used for automated surveillance of household and agricultural spaces enabling confirmatory detection and identification of this dangerous plant species.

## Abstract 2

Poison ivy (*Toxicodendron radicans*) is a noxious weed that grows throughout North America and induces terrible rashes on contact. Using a portable Raman device, we identified these plants and differentiated them from other species with high accuracy.

## Graphical abstract

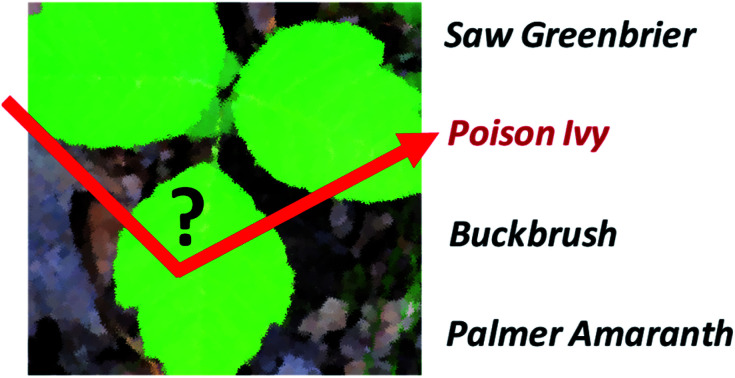

## Introduction

Poison ivy (*Toxicodendron radicans*) is a plant species that is broadly spread across North America.^1^ It produces urushiol that causes severe allergic reactions on skin including reddish inflammation, uncoloured bumps and blistering. Urushiol is a mixture of 1-(alkyl)- or 1-(alkenyl)-2,3-dihydroxybenzenes, where the alkyl/alkenyl group is C_15_–C_17_ saturated or unsaturated hydrocarbons.^2^ Once adsorbed by the skin, urushiol is captured by immune system dendritic cells that migrate to lymph nodes where they present it to T-lymphocytes.^3^ Activated T-lymphocytes migrate to the skin areas rich with urushiol where, through the release of cytokines, they develop an autoimmune skin inflammation known as urushiol-induced contact dermatitis. The immune response typically develops within hours to days, which facilitates unknowing spread of the urushiol inside the house or through personal belongings, such as cell phones and clothes.^1^ Thus, a person may continuously come in contact with urushiol present on door knobs, desks, *etc.*, substantially extending the length of time of the rash.

The problem of poison ivy can be solved by timely detection, identification, and elimination of plants from publicly accessible areas. Such detection and identification typically require substantial botanical expertise that is not possessed by most people. The alternative is an image recognition software, which has been steadily improving since the 2000's.^4^ In particular, the yearly PlantCLEF competition has pushed the field of species image recognition: between 2007 and 2017, over 10 000 species were added to the dataset with the best tools able to classify 84% of samples.^5^ Additionally, a variety of mobile applications have been developed for plant species identification.^6,7^ Although such image recognition approaches enable fast species identification, they are not always accurate. Plant genotyping can overcome this limitation, enabling highly accurate species identification. However, these methods are destructive, time-consuming and labor-intensive.

Our group recently showed that Raman spectroscopy (RS) can be used for label-free, non-invasive and non-destructive identification of six different corn varieties.^8^ This identification is based on unique chemical composition of these varieties that can be probed by Raman spectroscopy, an analytical technique that is based on inelastic light scattering.^9^ Our group also showed that in addition to identification of plant varieties, RS can be used for confirmatory diagnostics of fungal diseases on corn, wheat and *Sorghum*.^10,11^ We further showed that RS could be also used for detection of viral diseases of wheat and rose, as well as the bacteria that cause Huanglongbing (HLB or Citrus Greening) on citrus trees.^12–14^ These diagnostics are based on detection of pathogen-induced changes in structure and composition of plant molecules. Such changes are unique for each species of pathogens.^9^

In this work, we investigate the potential of RS for confirmatory, non-invasive and non-destructive identification of poison ivy. Such identification is important in both natural forest and agricultural ecosystems. Using a hand-held Raman spectrometer, we collected spectra from leaves of poison ivy, as well as leaves of several wild (tree: water oak; shrub: buckbrush; ivy: saw greenbrier; grasses: palmer amaranth and white crownbeard) plant species grown in a forest in College Station, TX. We also compared Raman spectrum of poison ivy to the spectra collected from nine different cultivated species, including orange, grapefruit, roses, wheat, peanut, corn, marijuana, hemp, and potato. Lastly, we used partial least square discriminant analysis (PLS-DA) to discriminate between Raman spectra from poison ivy and other species.

## Experimental

### Plants

Poison ivy (*Toxicodendron radicans*), water oak (*Quercus nigra*) white crownbeard (*Verbesina virginica*), buckbrush (*Symphoricarpos orbiculatus*) and saw greenbrier (*Smilax bona-nox*) are naturally occurring species in the forest/park area in College Station, TX; hemp and marijuana plants were grown at Evergreen Enterprises LLC located in Denver, CO; leaves of orange and grapefruit trees were collected from plants grown in RioFarms, Monte Alto, TX and Texas A&M University-Kingsville Citrus Center, Weslaco, TX. Potato, roses (The Double Knock Out®), palmer amaranth and corn plants were grown in greenhouses located in College Station, TX; wheat plants were grown in a greenhouse in Amarillo, TX, whereas peanuts plants were grown in a greenhouse in Stephenville, TX.

All analyzed samples were fresh live plants. No special sample preparation was made.

### Raman spectroscopy

Raman spectra were taken with a hand-held Resolve Agilent spectrometer equipped with 830 nm laser source (beam diameter ∼ 2 mm). The following experimental parameters were used for all collected spectra: 1 s acquisition time, 495 mW power, and baseline spectral subtraction by device software. From two to four spectra were collected from each leaf on the adaxial side of the leaf. In total, 20 to 131 surface spectra from each plant species were collected. Spectra shown in the manuscript are raw baseline corrected, without smoothing.

### Multivariate data analysis

All Raman spectra were imported into MATLAB and analyzed with the add-on PLS_Toolbox (Eigenvector Research Inc.). Spectra were normalized to total area and the mean offset was removed at each wavenumber before use in partial least squares discriminant analysis (PLS-DA).

## Results and discussion

**Fig. 1:**
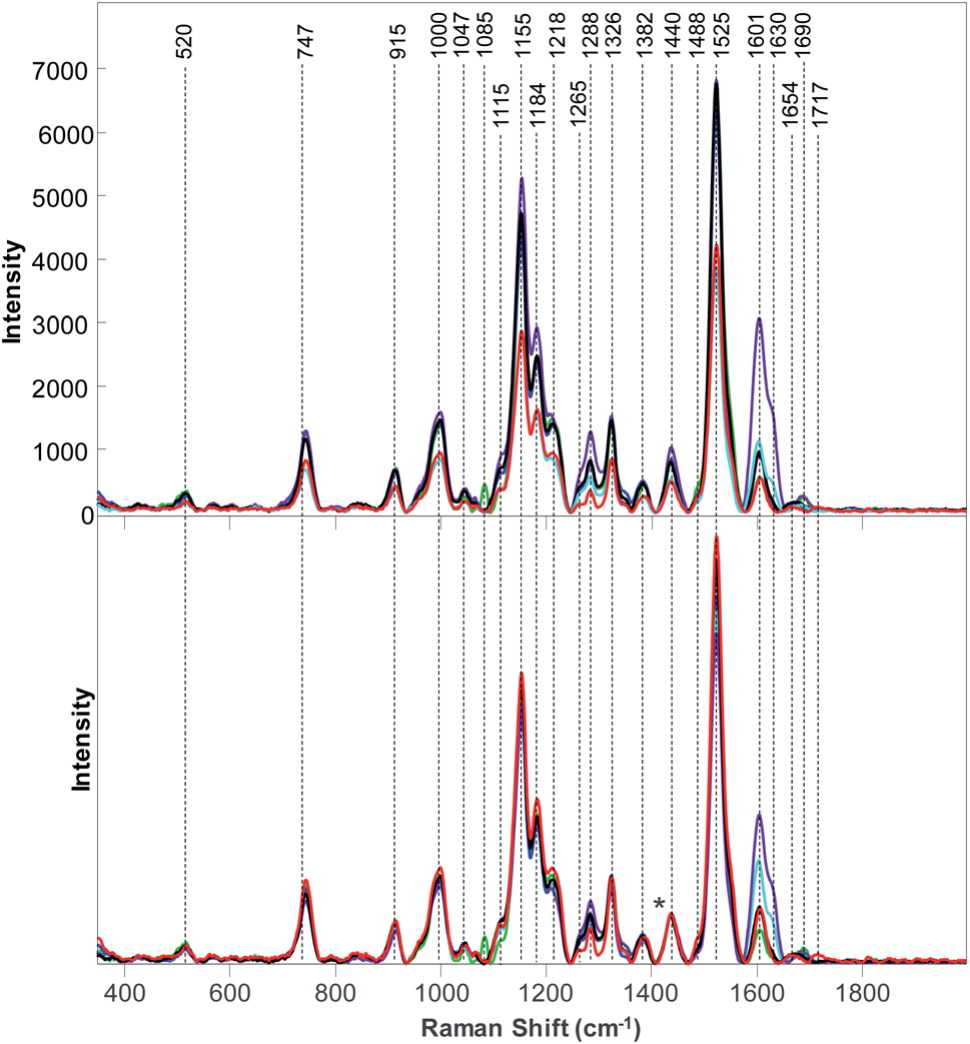
Baseline-corrected (top) and area normalized (bottom) Raman spectra collected from poison ivy (red), palmer amaranth (green), water oak (blue), white crownbeard (marine), buckbrush (purple) and saw greenbrier (black).

Raman spectra collected from six different wild plant species exhibited vibrational bands that could be assigned to cellulose (520, 915, 1047, 1115, 1326 cm^−1^), pectin (747 cm^−1^), carotenoids (1000, 1155, 1525 cm^−1^), phenylpropanoids (1047, 1326, 1601–1630 cm^−1^), xylan (1184 and 1218 cm^−1^), protein (1000 and 1654 cm^−1^), as well as to aliphatic (1218, 1288, 1382, 1440, 1488 cm^−1^) and carbonyl/ester (1690–1717 cm^−1^) groups (Table 1). We found that intensities of these bands vary between both non-normalized and normalized spectra collected from different plant species. For instance, Raman spectra collected from buckbrush leaves exhibited the most intense 1601–1630 cm^−1^ bands (phenylpropanoids), while intensity of these bands was the lowest in the spectrum of palmer amaranth (Fig. 1). We have also found that palmer amaranth showed a unique vibrational band at 1085 cm^−1^, which was not evident in Raman spectra of any other plant species. Although buckbrush, palmer amaranth and white crownbeard exhibited an intense band at 1690 cm^−1^, other species did not have a band at this frequency. We have also found that Raman spectra collected from leaves of poison ivy exhibited a unique band at 1717 cm^−1^, which was not evident in the Raman spectra of other plant species. Raman spectra collected from this plant species appeared to have the highest intensity of carotenoids (1000, 1155, 1525 cm^−1^) relative to five other wild plant species analyzed in our work. These results demonstrate that wild plant species have distinctly different Raman fingerprints that can be used for their identification. It should be noted that spectroscopic analysis of poison ivy reported in this manuscript was performed in April. Future studies are required to reveal possible changes in the spectra of this dangerous plant species that could take place in summer and fall. It is also important to reveal possible differences between spectroscopic signatures of poison ivy that was grown in different geographic locations to make such Raman-based identification of poison ivy robust and reliable.

**Table 1:** Vibrational bands and their assignments for leaves

Band	Vibrational mode	Assignment
520	*ν*(C–O–C) glycosidic	Cellulose^15^
747	*γ*(C–O–H) of COOH	Pectin^16^
915	*ν*(C–O–C) in plane, symmetric	Cellulose, phenylpropanoids^15^
1000	*ν*_3_ (C–CH_3_ stretching) and phenylalanine	Carotenoids, proteins^17,18^
1047	*ν*(C–O) + *ν*(C–C) + *δ*(C–O–H)	Cellulose, phenylpropanoids^15^
1085	*ν*(C–O) + *ν*(C–C) + *δ*(C–O–H)	Carbohydrates^19^
1115	*ν*_sym_(C–O–C), C–OH bending	Cellulose^15^
1155	C–C stretching; *ν*(C–O–C), *ν*(C–C) in glycosidic linkages, asymmetric ring breathing	Carotenoids,^20^ cellulose^21^
1184	*ν*(C–O–H) next to aromatic ring + *σ*(CH)	Xylan^22,23^
1218	*δ*(C–C–H)	Aliphatic,^24^ xylan^22^
1265	Guaiacyl ring breathing, C–O stretching (aromatic)	Phenylpropanoids^25^
1288	*δ*(C–C–H)	Aliphatic^24^
1326	*δ*CH_2_ bending vibration	Cellulose, phenylpropanoids^26^
1382	*δ*CH_2_ bending vibration	Aliphatic^24^
1440	*δ*(CH_2_) + *δ*(CH_3_)	Aliphatic^24^
1488	*δ*CH_2_ bending vibration	Aliphatic^24^
1525	–C=C– (in plane)	Carotenoids^27,28^
1601–1630	*ν*(C–C) aromatic ring + *σ*(CH)	Phenylpropanoids^29,30^
1654	Amide I	Proteins^31^
1690–1717	*ν*(C=O)	Carboxyl/ester groups^32^

Next, we used PLS-DA to determine whether RS can be used for the qualitative identification of these wild plant species based on spectroscopic signatures collected from their leaves. Our results show that poison ivy can be distinguished from all other analyzed plant species with 100% accuracy, Table 2. Moreover, PLS-DA results demonstrate that all wild species except buckbrush can be identified with 100% accuracy (accuracy of buckbrush identification was 98.2%). These findings suggest that RS can be used for highly accurate detection and identification of wild plant species, including poison ivy.

**Table 2:** Accuracy of classification by PLS-DA wild plant species

	Correct, %	Palmer amaranth	Poison ivy	Water oak	White crownbeard	Buckbrush	Saw greenbrier
Palmer amaranth	100	30	0	0	0	0	0
Poison ivy	100	0	52	0	0	1	0
Water oak	100	0	0	52	0	0	0
White crownbeard	100	0	0	0	55	0	0
Buckbrush	98.2	0	0	0	0	55	0
Saw greenbrier	100	0	0	0	0	0	52

**Fig. 2:**
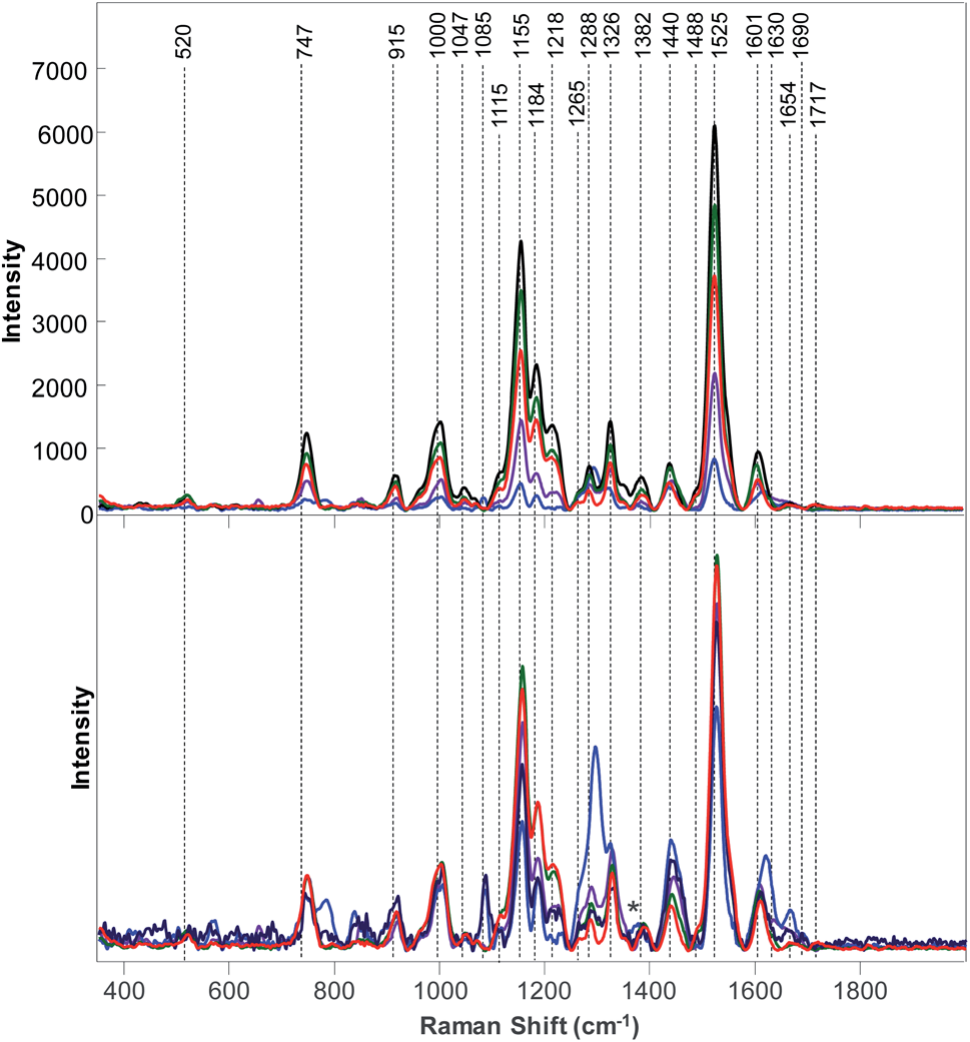
Baseline-corrected (top) and area normalized (bottom) Raman spectra Raman collected from poison ivy (red), orange (green), marijuana (blue), roses (black), and peanuts (purple).

**Fig. 3:**
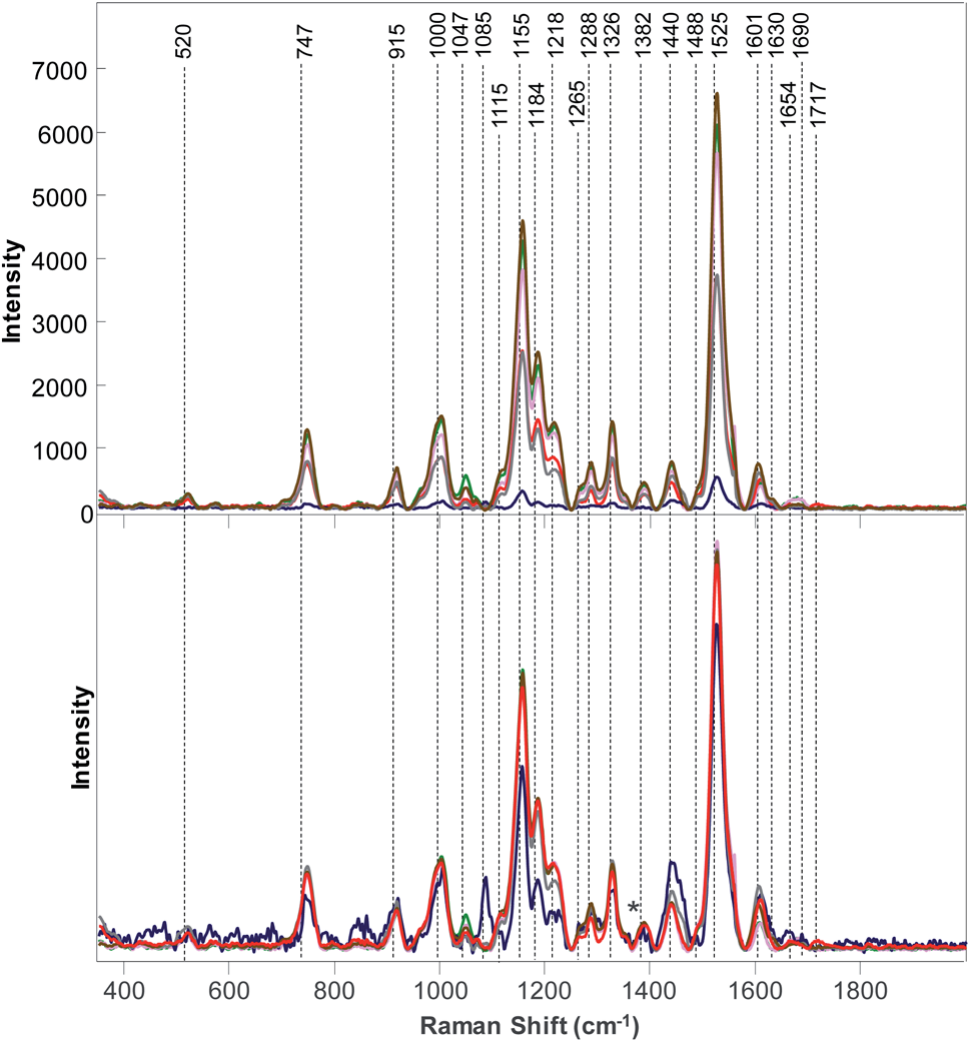
Baseline-corrected (top) and area normalized (bottom) Raman spectra Raman collected from poison ivy (red), wheat (green), potato (pink), hemp (marine blue), grapefruit (grey) and corn (brown).

The question to ask is whether RS can be also used to distinguish poison ivy in agricultural ecosystems. To answer this question, we collected Raman spectra from nine different cultivated plant species, Fig. 2 and 3. We have found that leaves of cultivated plants exhibit similar spectroscopic profiles. The obtained spectra were dominated by vibrational bands that could be assigned to the same classes of scaffold molecules that were present in the leaves of wild species. Only Raman spectra collected from marijuana plants exhibited substantial differences in the observed vibrational bands, which could be assigned to delta-9-tetrahydrocannabinolic acid (THCA), the precursor to the psychoactive compound present in these plants.^33^

We have also found that intensities of vibrational bands that could be assigned to cellulose, pectin, carotenoids, phenylpropanoids, xylan, protein, as well as aliphatic and carbonyl/ester groups varied a lot from one plant species to another. For instance, intensities of carotenoid vibrations were found to be the most intense in the spectra of orange, potato and corn, whereas the intensities of these vibrations have been found to be substantially less intense in the Raman spectra of marijuana and hemp. Some of the plant species, such as roses, peanuts and hemp exhibited an intense amide I vibration that can be assigned to proteins, whereas other spectra collected from other plant species did not exhibit an intense vibration in this spectral region. It should be noted that the discussed above 1717 cm^−1^ band that has been observed in the spectrum of poison ivy was not evident in any of the spectra of cultivated plant species. This suggests that this band can be used to identify this dangerous plant species based on the spectroscopic fingerprint of its leaves.

We used PLS-DA to enable quantitative identification of poison ivy among all analyzed cultivated plants. Our results demonstrate that poison ivy can be distinguished from all cultivated plant species with 100% accuracy, Table 3. Moreover, PLS-DA results demonstrate that wheat, peanuts, marijuana, potato, and hemp can be identified with 100% accuracy, whereas roses, oranges, grapefruit and corn can be identified with 97.7%, 97.9%, 97.9% and 98.4% accuracy, respectively. These findings suggest that RS can be used for highly accurate detection and identification of poison ivy agricultural ecosystems.

**Table 3:** Accuracy of classification by PLS-DA cultivated plant species

	Correct, %	Wheat	Peanuts	Marijuana	Roses	Potato	Poison ivy	Orange	Hemp	Grapefruit	Corn
Wheat	100	20	0	0	0	0	0	0	0	0	0
Peanuts	100	0	50	0	2	0	0	0	0	0	0
Marijuana	100	0	0	20	0	0	0	0	0	0	0
Roses	97.7	0	0	0	131	0	0	0	0	0	0
Potato	100	0	0	0	0	24	0	0	0	0	0
Poison ivy	100	0	0	0	0	0	57	0	0	0	0
Orange	97.9	0	0	0	1	0	0	47	0	1	0
Hemp	100	0	0	0	0	0	0	0	22	0	0
Grapefruit	97.9	0	0	0	0	0	0	1	0	47	0
Corn	98.4	0	0	0	0	0	0	0	0	0	63

## Conclusions

Our results demonstrate that RS can be used for confirmatory, non-invasive and non-destructive identification of poison ivy among both wild and cultivated plant species. These findings also suggest that RS can be used for automated identification of this dangerous plant in both forest and agricultural ecosystems. One can envision that coupling of RS with robotic platform or a UAV will enable automated surveillance of household and agricultural spaces enabling confirmatory detection and identification of this dangerous plant species. Rapid development of portable Raman spectrometers also offers a possibility to make a pocket device that any farmer or property owner can have in their possession.^34,35^ Once equipped with a spectroscopic library of plant species, such units can be used for daily inspection of plants in parks, forests and agricultural ecosystems.

## Conflicts of interest

There are no conflicts to declare.

## Supplementary Material
